# Silicon Supply to Barley Reduces Leaf Diseases Intensity and Increases Flexibility of Fungicide Interventions

**DOI:** 10.3390/plants15111654

**Published:** 2026-05-28

**Authors:** Anderson Eduardo Brunetto, Jaqueline Hagn, Leandro José Dallagnol

**Affiliations:** Departamento de Fitossanidade, Faculdade de Agronomia Eliseu Maciel, Universidade Federal de Pelotas, Pelotas 96010-900, RS, Brazil; brunettoagronomo@hotmail.com (A.E.B.); jaquelinehagn@gmail.com (J.H.)

**Keywords:** *Hordeum vulgare*, disease control, integrated disease management (IDM), fungicide programs, sustainable agriculture

## Abstract

Silicon (Si) has been recognized as a beneficial element in plant disease resistance; however, its role under field conditions in barley remains insufficiently explored. This study evaluated the effects of soil-applied Si and its interaction with cultivars and fungicide programs on foliar disease control and yield components in barley over four growing seasons. Two field experiments were conducted: Study I assessed the interaction between Si supply, cultivars, and fungicide application, while Study II evaluated different fungicide programs under Si supply. Silicon application consistently reduced disease severity and the area under the disease progress curve for powdery mildew and leaf spots across cultivars and seasons. The combined use of Si and fungicides resulted in the greatest disease reduction. Cultivar BRS Cauê, showing higher partial resistance than BRS Brau, showed greater responsiveness to Si, which was associated with higher foliar Si concentration. Fungicide programs P2 [sprays at growth stage (GS) 12, GS26, GS37, and GS61] and P3 (sprays at GS12, GS31, and GS 61) were the most effective for disease control and under Si supply. Fungicide program P3, despite one fewer application, achieved disease control comparable to P2, suggesting the potential for optimizing fungicide use under Si supply. Grain yield increases closely followed reductions in disease intensity, with the highest yields observed under combined Si supply and optimized fungicide programs. These findings demonstrate that Si contributes to disease reduction and yield improvement and may serve as a key component of integrated and sustainable disease management in barley.

## 1. Introduction

Barley (*Hordeum vulgare* L.) is one of the major cereal crops in the world and is widely used for both human and animal consumption [1]. For human use, barley can be consumed directly or processed into malt for the brewing industry [2]. In Brazil, most of the barley produced is designated for beer production [3].

Malt quality is directly affected by barley grain attributes [4], and the occurrence of fungal diseases is among the main threats limiting yield potential and grain qualities [5,6]. Powdery mildew (*Blumeria graminis* f. sp. *hordei*), net blotch (*Pyrenophora teres*), and spot blotch (*Bipolaris sorokiniana*) are the diseases most commonly associated with higher damages in barley crop [6].

For the control of barley diseases, the use of integrated disease management (IDM) is crucial to achieve optimal results. IDM includes several strategies, such as crop rotation, resistant cultivars, the use of healthy seeds, balanced nutrition, and fungicide application [3,6]. Fungicides are widely used for barley disease control and represent an effective management tool; however, they do not provide complete (100%) control of all diseases, particularly when applied under conditions of high disease pressure [7,8,9]. In adequate IDM, practices that reduce disease pressure slow the epidemic development and increase the effectiveness of other control measures, such as fungicides. An option to slow down epidemics in plants is the use of silicon [10].

Silicon (Si) is a beneficial element that enhances plant resistance to several diseases in both monocot and dicot plants, regardless of the type of parasitism established by the pathogen [10]. Among winter cereals, wheat is the crop where reductions in disease severity promoted by Si have been associated with increase in grain yield and quality [11,12,13]. In barley, Si has been shown to increase plant resistance to powdery mildew, Fusarium head blight (*Fusarium* spp.), and spot blotch [14,15,16,17]. Regarding spot blotch, Si-supplied plants exhibited improved performance even under pathogen attack, enhancing fungicide efficacy, particularly toward the end of residual control period [16,17]. However, under field conditions, available information on the use of Si in biotic stress management in barley is still scarce.

Considering the facts mentioned above, it is hypothesized that the use of Si in field-grown barley may represent an alternative approach to complement IDM. To test this hypothesis, two studies were conducted: (i) the first study evaluated the effect of Si in association with foliar fungicide application on the control of foliar diseases; and (ii) the second study assessed the potential to reduce or reposition fungicide applications in Si-supplied plants aiming at disease control in barley.

## 2. Results

### 2.1. Study I

**Figure 1:**
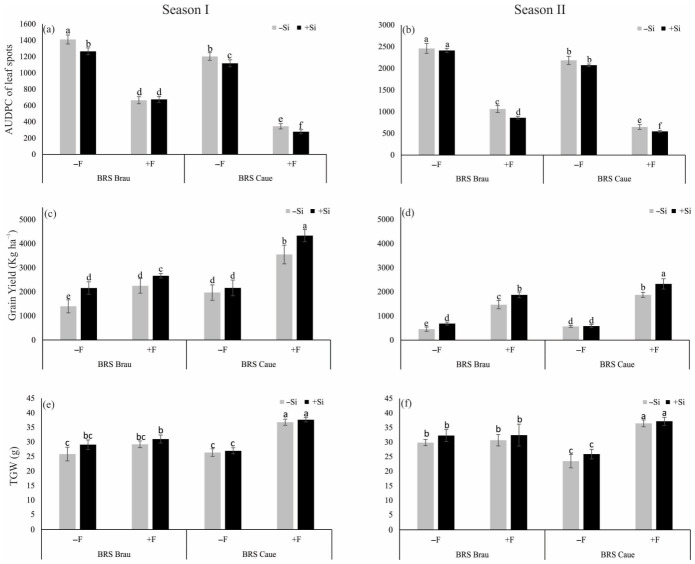
Effect of silicon (Si) supply and fungicide application on disease development and yield components of barley cultivars BRS Brau and BRS Cauê in the season I (**a**,**c**,**e**) and season II (**b**,**d**,**f**). (**a**,**b**) Area under the disease progress curve (AUDPC) of leaf spots; (**c**,**d**) grain yield (kg ha^−1^); and (**e**,**f**) thousand grain weight (TGW, g). Treatments consisted of the absence (−Si) or presence (+Si) of silicon combined with the absence (−F) or presence (+F) of fungicide application. Data are presented as mean ± standard error. Different letters indicate significant differences among treatments according to Tukey’s test (*p* ≤ 0.05).

This study evaluated the effect of Si associated with fungicide spraying (with or without fungicide treatment) in two barley cultivars (BRS Brau and BRS Cauê) over two growing seasons. Silicon supply reduced AUDPC in both cultivars, particularly in the absence of fungicide, whereas fungicide application resulted in the greatest reduction in disease intensity in both seasons (I and II) (Figure 1a,b and Figure S1).

The combined use of Si and fungicide further decreased AUDPC, especially for BRS Cauê (Figure 1a,b). Grain yield increased with both Si and fungicide, with the highest values observed under Si supply and fungicide treatment, especially for BRS Cauê (Figure 1c,d). Thousand grain weight showed a similar response pattern, although differences among treatments were less pronounced; however, the highest values were recorded in plants of cultivar BRS Cauê supplied with Si and treated with fungicide in both seasons (Figure 1e,f).

No significant interaction between soil amendments and cultivars was observed for leaf Si concentration. Soil amendment with calcium silicate resulted in increases of 84.5% (from 4.52 mg kg^−1^ of dry matter in −Si plants to 8.34 mg kg^−1^ in +Si plants) and 47.7% (from 4.04 to 5.97 mg kg^−1^ of dry matter) in foliar Si concentration compared with extra-fine limestone in the seasons I and II, respectively. In the presence of silicon fertilization, the cultivar BRS Cauê showed higher foliar Si concentrations than BRS Brau, with increases of 15% (from 7.65 to 9.02 mg kg^−1^ dry matter) and 27% (from 5.03 to 6.93 mg kg^−1^ dry matter) in seasons I and II, respectively.

### 2.2. Study II

**Figure 2:**
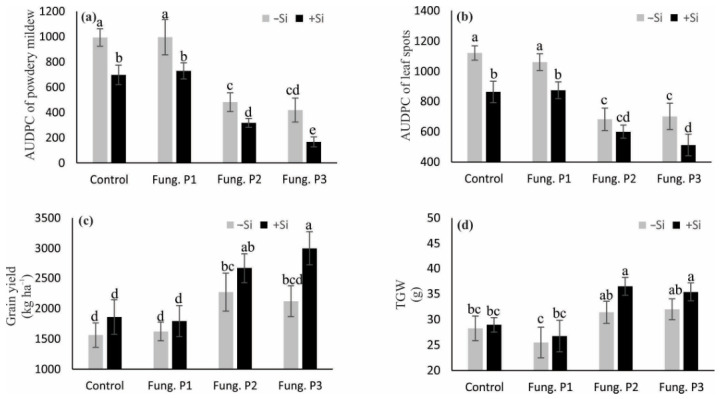
Effect of silicon (Si) supply and fungicide programs on disease development and yield of barley cultivar BRS Cauê during the first growing season of the Study II. (**a**) Area under the disease progress curve (AUDPC) of powdery mildew; (**b**) AUDPC of leaf spots; (**c**) grain yield (kg ha^−1^); and (**d**) thousand grain weight (TGW, g). Treatments consisted of the absence (−Si) or presence (+Si) of Si combined with different fungicide programs [control, fungicide program 1 (P1), program 2 (P2), and program 3 (P3)]. Data are presented as mean ± standard error. Different letters indicate significant differences among treatments according to Tukey’s test (*p* ≤ 0.05).

**Figure 3:**
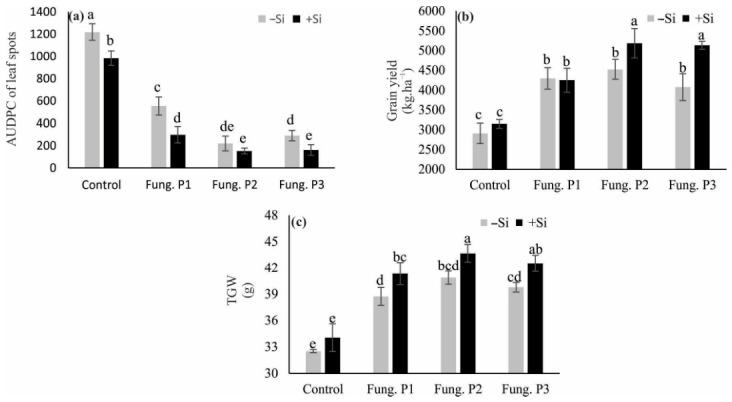
Effect of silicon (Si) supply and fungicide programs on leaf spot development and yield of barley cultivar BRS Cauê during the second growing season of the Study II. (**a**) Area under the disease progress curve (AUDPC) of leaf spots; (**b**) grain yield (kg ha^−1^); and (**c**) thousand grain weight (TGW, g). Treatments consisted of the absence (−Si) or presence (+Si) of Si combined with different fungicide programs (control, fungicide program 1 (P1), program 2 (P2), and program 3 (P3)). Data are presented as mean ± standard error. Different letters indicate significant differences among treatments according to Tukey’s test (*p* ≤ 0.05).

This study, conducted over two growing seasons, was designed to evaluate the effect of Si supply in a barley cultivar with a greater capacity to accumulate the element (BRS Cauê), as well as its potential to reduce or reposition fungicide applications during the crop cycle. Silicon supply and fungicide programs significantly affected disease development and yield components (Figure 2, Figure 3 and Figure S2). Powdery mildew was recorded only in the first year of the experiment, and its severity increased progressively throughout crop development (Supplementary Figure S2). However, Si application significantly reduced powdery mildew severity. Powdery mildew severity was approximately 60% in the plants without Si and decreased to about 40% in Si-supplied plants, representing a reduction of 32%. This effect was also reflected in the AUDPC, which was reduced by 30% in Si-supplied plants compared with non-supplied plants under the control treatment (Figure 2a).

Leaf spots severity followed a similar pattern to that of powdery mildew, increasing with crop development (Supplementary Figure S2). Silicon-supplied plants showed a reduced leaf spots severity, with significant reductions compared with plants not supplied with Si in both seasons of the study (Supplementary Figure S2). This effect was also reflected in the AUDPC values (Figure 2b and Figure 3a), where Si consistently reduced disease accumulation under the control treatment.

The AUDPC for powdery mildew and leaf spots was significantly influenced by both fungicide treatments and Si supply (Figure 2a,b and Figure 3a). In the first year of the study, the control and fungicide program 1 showed the highest AUDPC values, whereas fungicide programs 2 and 3 significantly reduced disease intensity (Figure 2a,b). Silicon further decreased AUDPC across all fungicide treatments, with the lowest values observed when Si was combined with fungicide program 3, demonstrating an additive effect between Si and fungicide-based management. In the second year of the study, all fungicide programs reduced AUDPC, with the lowest values observed in Si-supplied plants compared to non-supplied plants, except for fungicide program 2 (Figure 3a).

Grain yield was significantly affected by fungicide programs and Si supply (Figure 2c and Figure 3b). In the absence of fungicides or under fungicide program 1, Si did not result in significant yield increases. However, under fungicide programs 2 and 3, Si significantly enhanced yield, with the highest yield observed when Si was combined with fungicide program 3. This corresponds to yield increases of 92% and 75% in the first and second years, respectively, compared with the untreated control without Si.

TGW was also positively influenced by Si supply and fungicide treatments (Figure 2d and Figure 3c). Silicon significantly increased TGW under fungicide programs 2 and 3. In contrast, lower TGW values were observed in the control in both years and under fungicide program 1, particularly in the first year.

No significant interaction was observed between soil amendments and fungicide programs for leaf Si concentration. Soil amendment with calcium silicate, compared with extra-fine limestone, increased foliar Si concentrations by 66% (from 5.7 mg kg^−1^ dry matter in −Si plants to 9.5 mg kg^−1^ in +Si plants) and 96% (from 5.9 to 11.6 mg kg^−1^ dry matter) in seasons I and II, respectively, of the Study II.

## 3. Discussion

The results of this study consistently demonstrate that calcium silicate supply, a source of soluble Si, reduced disease severity and AUDPC across cultivars, fungicide programs, and growing seasons, confirming its role as an important component of barley disease management. The observed reduction in AUDPC under field conditions corroborates previous greenhouse studies showing that Si decreases the severity of powdery mildew and spot blotch [14,16,17], and further indicates that these effects translate into increased grain yield under field conditions.

In Study I, although both cultivars showed reduced AUDPC with calcium silicate supply, BRS Cauê exhibited the greatest benefit, which may be associated with higher foliar accumulation of Si. Moreover, despite the relatively modest difference, BRS Cauê showed lower AUDPC than BRS Brau, a condition that may have enhanced the effectiveness of Si. This observation is consistent with previous studies reporting that cultivars with higher levels of partial resistance tend to derive greater disease reduction from Si application [11,12,18,19,20,21,22]. In this context, the use of more resistant cultivars can enhance the effectiveness of chemical disease control [8,23], which can be potentiated by suppling Si. This pattern was also observed in the present study, where the greatest reduction in AUDPC with the combined application of Si source and fungicide occurred in BRS Cauê. This interaction is especially relevant in the context of integrated disease management, where combining control strategies can enhance efficacy while reducing reliance on single approaches [24].

The combined application of Si source amendment in the soil and fungicide sprays improved barley disease control, indicating a complementary interaction between these management strategies and corroborating previous findings in other crops. In rice, blast control achieved with Si was comparable to that obtained with the fungicide benomyl, with the greatest reduction in disease severity observed when soil Si fertilization was combined with fungicide applications [25,26]. Furthermore, in Si-supplied plants, reduced fungicide doses provided levels of blast disease control similar to those achieved with full doses in the absence of Si [27]. In sugarcane, Si application in the form of organosilicon, in combination with copper hydroxide, reduced the leaf scald index even at lower fungicide doses, indicating that Si may help reduce copper inputs while maintaining adequate disease control [28].

Although these studies suggest that Si supply may allow reduce fungicide doses, there is evidence that sublethal doses can increase the risk of pathogen resistance to active ingredients, particularly for site-specific fungicides [29]. Therefore, optimizing the timing and positioning of fungicide applications may represent a more robust strategy to reduce the total volume of fungicide applied per growing season. This argument is supported by the results of Study II, which evaluated different fungicide programs. Despite the clear effect of Si, fungicide programs, particularly P2 and P3, remained the most effective treatments for reducing disease severity, confirming their central role in disease management under high disease pressure. However, the additional reduction in AUDPC observed when Si was combined with these programs indicates an additive effect, suggesting that Si and fungicides act through complementary mechanisms.

In this context, program P3, which included one fewer fungicide application than P2, provided disease control comparable to P2, particularly in Si-supplied plants. This finding highlights the potential for repositioning fungicide applications or choosing better fungicide combination in each application, reducing the number of sprays while maintaining disease control at acceptable levels. In contrast, in program P1, in which no fungicide application was performed at the GS12 stage, disease progress was more intense, indicating that applications starting only at GS26 were insufficient to achieve satisfactory control of powdery mildew and leaf spot diseases. Notably, even under program P1, Si-supplied plants exhibited improved disease control, which is likely associated with Si-induced changes in plant resistance components. These changes may reduce the rate of disease progress, thereby enhancing fungicide effectiveness, as previously reported [16,17,20].

The increase in grain yield observed across treatments in Studies I and II closely followed the reductions in disease intensity, reinforcing the strong relationship between disease control and yield. The highest yields, particularly in the cultivar BRS Cauê, were consistently associated with treatments combining Si with well-positioned fungicide applications, as observed in programs P2 and P3, indicating that yield gains were maximized when disease pressure was effectively minimized. This pattern suggests that Si contributes to yield primarily through its role in disease suppression, by preserving green leaf area and maintaining photosynthetic capacity during grain filling, as previously reported [16,17]. However, in cultivar BRS Brau, in Study I, a substantial increase in grain yield was observed, particularly in plants without fungicide application, even under conditions of only a modest reduction in AUDPC. This finding suggests that additional benefits associated with calcium silicate supply, beyond disease severity reduction, may have contributed to this response. However, the data obtained in the present study do not allow us to infer which specific benefits may have been involved.

In this context, the effects on TGW, although less pronounced and more variable across treatments, followed a similar trend to yield, indicating that yield increases were driven mainly by grain size rather than grain number. Nevertheless, the tendency for higher TGW under Si supply suggests an additional physiological benefit, possibly related to improved water use efficiency and delayed senescence under biotic stress, as previously reported in Si-treated cereals [30].

The consistency of Si effects across the four growing seasons (Study I and II) further highlights its robustness under variable environmental conditions. Seasonal variation is a major factor influencing disease dynamics in cereal crops, particularly in barley and wheat, where temperature and humidity strongly affect diseases development [31,32]. The stable reduction in AUDPC and the associated yield benefits observed here suggest that Si can contribute to buffering these environmental effects, thereby enhancing crops resilience. In addition, the beneficial effects of Si on alleviating abiotic stresses and improving the efficiency of chemical fertilizer use, particularly phosphorus fertilization, have also been reported [33]. These findings further indicate that Si may play an important role in enhancing plant resistance to multiple stresses under field conditions, where biotic stresses are frequently compounded by adverse environmental factors.

Overall, the results indicate that Si plays an important role in barley production systems by reducing disease progress (lower AUDPC), enhancing the effectiveness of fungicide programs, and increasing grain yield. These findings support the inclusion of calcium silicate as source of Si in the integrated and sustainable disease management strategies in barley.

## 4. Material and Methods

### 4.1. Field Characteristics

Two sets of experiments were conducted over four growing seasons (four years) at the experimental area of the Federal University of Pelotas, located in the municipally of Capão do Leão (31°80′ S, 52°50′ W), Brazil. The first set of experiments [two growing seasons (two years)], designated Study I henceforth, aimed to evaluate whether calcium silicate, source of soluble Si, affects the intensity of barley diseases. The second set [two growing seasons (two years)], designated Study II henceforth, aimed to assess fungicide application programs for controlling foliar diseases in Si-supplied plants. All experiments were conducted under a monoculture system with a barley–soybean sequence.

The set of experiments of the Study I was conducted in an area where experiments with Si were already ongoing. Accordingly, blocks were designated as +Si for calcium silicate-treated blocks and −Si for limestone-treated blocks. The −Si blocks presented the following chemical characteristics: pH in water (1:1) = 5.8; P (Mehlich) = 22 mg dm^−3^; K = 99 mg dm^−3^; concentrations of calcium (Ca^2+^), magnesium (Mg^2+^) and aluminum (Al^3+^), potential acidity (H + Al^3+^), and effective cation exchange capacity (CEC) = 1.84, 1.18, <0.1, 1.55 and 10.4 cmol_c_·dm^−3^ respectively; base saturation = 85%; organic matter (*v*/*v*) = 2.8% and clay content (*v*/*v*) = 20%; and SMP index = 6.5. Available Si content, extracted with CaCl_2_ was 6.6 mg dm^−3^. The +Si blocks presented similar chemical characteristics, except for available Si content, extracted with CaCl_2_, which was 13.5 mg dm^−3^.

The set of experiments of the Study II was conducted in a new area adjacent to the first experimental site, where calcium silicate had never been applied. The soil presented the following chemical characteristics: pH in water (1:1) = 4.9; P (Mehlich) = 10.6 mg dm^−3^; K = 58 mg dm^−3^; concentrations of Ca ^2+^, Mg^2+^, Al^3+^, H + Al^3+^,and CEC = 3.1, 1.7, 0.6, 4.4, and 5.7 cmol_c_.dm^−3^ respectively; base saturation = 53%; organic matter (*v*/*v*) = 1.94% and clay content (*v*/*v*) = 25%; and SMP index = 6.0. Silicon available, extracted with CaCl_2_, was 6.0 mg dm^−3^.

### 4.2. Liming Sources and Rates

Calcium silicate (Agrosilício Plus^®^, Agronelli Insumos Agrícolas, Uberaba, Brazil), containing 10.5% Si, 25.0% Ca, and 6.0% Mg, was used as the Si source. To standardize the amounts of Ca and Mg supplied in the calcium silicate treatment, the control soil was amended with extra-fine limestone (Dagoberto Barcelos, Caçapava do Sul, Brazil), containing 26.5% Ca and 15.0% Mg.

In Study I, calcium silicate was applied at 2.6 and 2.5 t ha^−1^ in the +Si treatments for the first and second crop seasons, respectively, whereas extra-fine limestone was applied at 3.0 and 2.6 t ha^−1^ for the corresponding seasons. Application rates were determined based on soil chemical analysis to raise soil pH to 6.5. Both amendments were incorporated into the soil with a rotary hoe 30 days prior to sowing. Soil samples collected at sowing showed Si concentrations of 11.7 and 10.1 in the +Si plots, and 6.6 and 6.6 mg kg^−1^ in the −Si plots, in the first and second seasons, respectively.

In Study II, during the first season, calcium silicate or extra-fine limestone was incorporated at 4.0 t ha^−1^ to increase soil pH to 6.5. In the second season, both amendments were applied at 2.0 t ha^−1^. In both seasons, soil correctives were incorporated by harrowing 30 days before sowing. Soil samples collected at sowing showed Si concentrations of 11.4 and 14.7 in the +Si plots, and 6.0 and 6.0 mg kg^−1^ in the −Si plots, in the first and second seasons, respectively.

### 4.3. Plant Material and Cropping Management

The barley cultivars BRS Brau and BRS Cauê (Embrapa Trigo, Passo Fundo, Brazil) were used in the experiments. Both cultivars are moderately resistant to net blotch and susceptible to powdery mildew, brown spot, and Fusarium head blight [3].

Seed sowing was performed in July using a seeder (SHM, Semeato, Passo Fundo, Brazil) configured with nine rows spaced 0.17 m apart. Seeding density was adjusted to establish 300 plants m^−2^. At sowing, 450 kg ha^−1^ of NPK fertilizer (5% N, 20% P_2_O_5_, and 20% K_2_O) was applied. Nitrogen topdressing was performed with urea (150 kg ha^−1^; 45% N) at growth stage 31 (GS31) according to the Zadoks scale [34]. Weed control was carried out 35 days after sowing using iodosulfuron (Hussar^®^, Bayer, Leverkusen, Germany) at 80 g ha^−1^. Pest management was conducted with thiamethoxam + lambda-cyhalothrin (Engeo Pleno^®^, Syngenta, Basel, Switzerland) at 50 mL ha^−1^ to reduce aphid populations as needed.

### 4.4. Experimental Design and Treatments

Study I consisted of a 2 × 2 × 2 factorial arrangement in a randomized complete block design, with a split-split-plot structure, in which factors were assigned to main plots, subplots, and sub-subplots, totaling eight treatments with four replicates. Each experimental plot measured 10 m^2^ (2 × 5 m). The main plot was the two soil amendments [calcium silicate (+Si) or extra-fine limestone (−Si)]; the subplot was the two barley cultivars (BRS Brau and BRS Cauê); and the sub-subplots was the two fungicide regimes (treated or untreated). The experiment was conducted over two growing seasons.

Study II consisted of a 2 × 4 factorial arrangement in a randomized complete block design, with a split-plot structure, in which factors were assigned to main plots and subplots, totaling eight treatments with four replicates. Each plot measured 10 m^2^ (2 × 5 m). The main plot was the two soil amendments [calcium silicate (+Si) or extra-fine limestone (−Si)], the subplot was the four fungicide programs. The fungicide treatments were as follows: (i) control (no fungicide application); (ii) program 1—three applications at GS26, GS37, and GS61; (iii) program 2—four applications at GS12, GS26, GS37, and GS61; (iv) program 3—applications based on disease thresholds under natural infection, performed at GS12, GS31, and GS61 (Table 1). The fungicide was applied using a CO_2_ pressured sprayer kit, with four tip nozzles (TTJ60 11002; Teejet^®^, Heights, IL, USA), delivering 200 L ha^−1^. This experiment was conducted over two growing seasons.

### 4.5. Disease Assessment

The diseases observed during the four years of the experiments were powdery mildew, spot blotch, and net blotch. Powdery mildew occurred only in the first year of Study II. Disease severity was visually assessed on all leaves of 16 plants located in the central area of each plot and quantified using the diagrammatic scale [35]. Evaluations were conducted at seven-day intervals, beginning at the onset of symptoms and continuing until the hard-dough stage of grain development. For leaf spots and powdery mildew, severity data were used to calculate the area under the disease progress curve (AUDPC) for each treatment using the formula proposed by Shaner and Finney [36].

### 4.6. Grain Yield (GY) and Thousand Grain Weight (TGW)

Barley was manually harvested from an area of 3.4 m^2^ (five rows, 4 m in length) and threshed using a mechanical grain thresher (EDA, model TR Parcela, De Antoni S.A., Caxias do Sul, Brazil). Grain moisture content was determined from a representative subsample using a moisture meter (Gehaka Agri G600, Gehaka, São Paulo, Brazil). Grain yield was measured using a precision balance and adjusted to a standard moisture content of 13%. Thousand grain weight (TGW) was determined by weighing a sample of 1000 grains on an analytical balance (Shimadzu (Tokyo, Japan) model BL-3200H) and expressed in grams.

### 4.7. Determination of Si Concentration

Leaves from plants of the Studies I and II were sampled at the flowering stage (GS60), rinsed with deionized water, dried for 72 h at 70 °C, and ground using a mortar and pestle to pass through a 40-mesh screen. Foliar Si concentrations were determined through colorimetric analysis of 0.1 g of alkali-digested tissue [37].

### 4.8. Data Analysis

Prior to analysis of variance (ANOVA), data normality was assessed using the Shapiro–Wilk test. All variables were subjected to ANOVA, and when significant effects were detected, silicon treatments and cultivars were compared using Student’s *t*-test (*p* ≤ 0.05), while fungicide treatments were compared using Tukey’s test (*p* ≤ 0.05). Statistical analyses were performed using STATISTICA software 7.0.

## Figures and Tables

**Table 1:** Fungicide spray programs, commercial products (CP), active ingredients (AI), AI concentration (g L^−1^) of fungicides applied to barley plants, and corresponding phenological growth stages at application (timing).

Fungicide Treatment	Active Ingredient (AI)	AI	Timing ^1^
		g L^−1^	
Control	-	-	-
Program 1	(Propiconazole) + (Epoxiconazole + Pyraclostrobin + Fluxapyroxad)	(250) + (50 + 81 + 50)	2
	Fenpropimorph + (Cyproconazole + Azoxystrobin)	(750) + (80 + 200)	3
	(Tebuconazole + Trifloxystrobin) + Mancozeb	(100 + 200) + 750	4
Program 2	(Propiconazole)	(250)	1
	Propiconazole + (Epoxiconazole + Pyraclostrobin + Fluxapyroxad)	(250) + (50 + 81 + 50)	2
	Fenpropimorph + (Cyproconazole + Azoxystrobin)	(750) + (80 + 200)	3
	(Tebuconazole + Trifloxystrobin) + Mancozeb	(100 + 200) + 750	4
Program 3	Propiconazole + (Epoxiconazole + Pyraclostrobin + Fluxapyroxad)	(250) + (50 + 81 + 50)	1
	Fenpropimorph + (Cyproconazole + Azoxystrobin)	(750) + (80 + 200)	2/3 *
	(Tebuconazole + Trifloxystrobin) + Mancozeb	(100 + 200) + 750	4

^1^ Growth stages according to the Zadoks scale: 1—GS12 (30 days after emergence [DAE]; third leaf unfolded); 2—GS26 (48 DAE; five to seven tillers); 3—GS37 (70 DAE; flag leaf emerged); 4—GS61 (84 DAE; beginning of flowering). * Application performed between timings 2 and 3, at GS31 (64 DAE). Fungicides used: Propiconazole (Tilt^®^; Syngenta); Epoxiconazole + Pyraclostrobin + Fluxapyroxad (Ativum^®^, BASF, Ludwigshafen, Germany), Fenpropimorph (Versatilis^®^, BASF), Cyproconazole + Azoxystrobin (Priori X^®^, Syngenta); Tebuconazole + Trifloxystrobin (Nativo^®^, Bayer), Mancozeb (Unizeb Gold^®^, UPL, Mumbai, India).

## Data Availability

The original contributions presented in this study are included in the article. Further inquiries can be directed to the corresponding author.

## Supplementary Materials

The following supporting information can be downloaded at: https://www.mdpi.com/article/10.3390/plants15111654/s1, Figure S1. Leaf spot severity over time in barley plants of cultivars BRS Brau and BRS Cauê grown in the absence (−Si) (a,c) or presence (+Si) (b,d) of silicon supply and either untreated (−F) or treated (+F) with fungicide during seasons I (a,b) and II (c,d) of Study I. P1, P2, and P3 represent the fungicide programs. Figure S2. Leaf spot severity (a,b,e,f) and powdery mildew severity (c,d) over time in barley plants of cultivar BRS Cauê grown in the absence (−Si) (a,c,e) or presence (+Si) (b,d,f) of silicon supply and either untreated (−F) or treated (+F) with fungicide during seasons I (a–d) and II (e,f) of Study II. P1, P2, and P3 represent the fungicide programs. Table S1. Effect of silicon (Si) supply [absence (−Si) or presence (+Si)] and fungicide application [absence (−F) or presence (+F)] on the area under the disease progress curve (AUDPC), grain yield (kg ha^−1^) and thousand grain weight (TGW, g) of barley cultivars BRS Brau and BRS Cauê in the seasons I and II of the study I. Data are presented as mean. Table S2. Leaf silicon (Si) concentration in barley cultivars BRS Brau and BRS Cauê grown in the absence (−Si) or presence (+Si) of Si supply during seasons I and II of Study I. Data are presented as means. Table S3. Severity of powdery mildew and leaf spots in barley plants of cultivar BRS Cauê grown in the absence (−Si) or presence (+Si) of silicon (Si) during different growth stages (GS) in season I of Study II. Data are presented as means. Table S4. Effect of silicon (Si) supply [absence (−Si) or presence (+Si)] and fungicide application [absence (−F) or presence (+F)] on the area under the disease progress curve (AUDPC), grain yield (kg ha^−1^) and thousand grain weight (TGW, g) of barley BRS Cauê in the seasons I of the study II. Data are presented as mean. Table S5. Severity of powdery mildew and leaf spots in barley plants of cultivar BRS Cauê grown in the absence (−Si) or presence (+Si) of silicon (Si) during different growth stages (GS) in season II of Study II. Data are presented as means. Table S6. Effect of silicon (Si) supply [absence (−Si) or presence (+Si)] and fungicide application [absence (−F) or presence (+F)] on the area under the disease progress curve (AUDPC), grain yield (kg ha^−1^) and thousand grain weight (TGW, g) of barley BRS Cauê in the seasons II of the study II. Data are presented as mean.
