# Supplementary material for: Silicon Supply to Barley Reduces Leaf Diseases Intensity and Increases Flexibility of Fungicide Interventions
Source: Plants (Basel). 2026 May 28;15(11):1654. doi: 10.3390/plants15111654 (PMC13258808; doi:10.3390/plants15111654)
Supplement: Supplementary file 1 [file plants-15-01654-s001.zip › plants-4291272-supplementary.pdf]

STUDY I

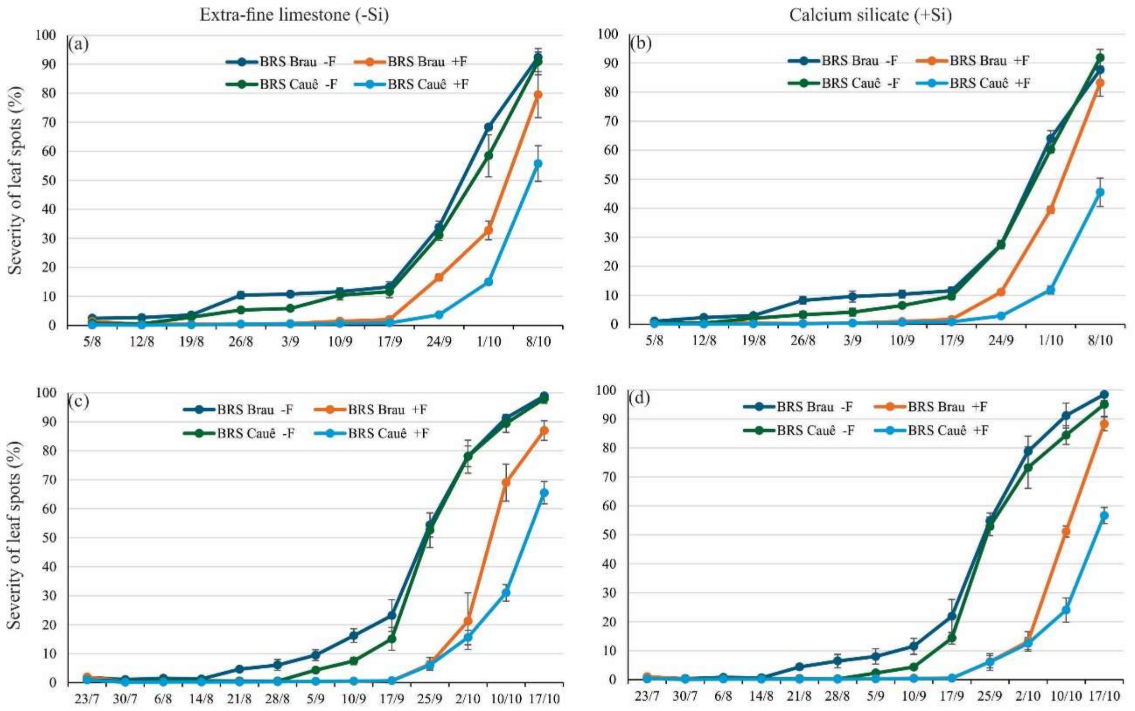

Figure S1. Leaf spot severity over time in barley plants of cultivars BRS Brau and BRS Cauê grown in the absence (−Si) (a and c) or presence (+Si) (b and d) of silicon supply and either untreated (−F) or treated (+F) with fungicide during seasons I (a and b) and II (c and d) of Study I. P1, P2, and P3 represent the fungicide programs.

Table S1. Effect of silicon (Si) supply [absence (−Si) or presence (+Si)] and fungicide application [absence (−F) or presence (+F)] on the area under the disease progress curve (AUDPC), grain yield (kg ha^{-1}) and thousand grain weight (TGW, g) of barley cultivars BRS Brau and BRS Cauê in the seasons I and II of the study I. Data are presented as mean.

	**AUDPC**							
	Season I				Season II			
	BRS Brau		BRS Cauê		BRS Brau		BRS Cauê	
	−F	+F	−F	+F	−F	+F	−F	+F
−Si	1410	664	1202	344	2458	1061	2181	645
+Si	1265	674	1117	274	2411	859	2070	535

	**Yield (Kg ha^{-1})**							
	Season I				Season II			
	BRS Brau		BRS Cauê		BRS Brau		BRS Cauê	
	−F	+F	−F	+F	−F	+F	−F	+F
−Si	1395	2246	1969	3548	450	1466	566	1873
+Si	2156	2664	2159	4328	682	1871	580	2326

	**TGW (g)**							
	Season I				Season II			
	BRS Brau		BRS Cauê		BRS Brau		BRS Cauê	
	−F	+F	−F	+F	−F	+F	−F	+F
−Si	25,8	29,2	26,3	36,8	29,8	30,7	23,5	36,45
+Si	29,1	31,0	26,9	37,6	32,2	32,4	25,9	37,2

Table S2. Leaf silicon (Si) concentration in barley cultivars BRS Brau and BRS Cauê grown in the absence (−Si) or presence (+Si) of Si supply during seasons I and II of Study I. Data are presented as means.

	Leaf Si concentration ($mg\ kg^{-1}$ dry matter)			
	Season I		Season II	
	BRS Brau	BRS Cauê	BRS Brau	BRS Cauê
−Si	4.15	4.90	3.25	4.83
+Si	7.65	9.03	5.03	6.93

STUDY II

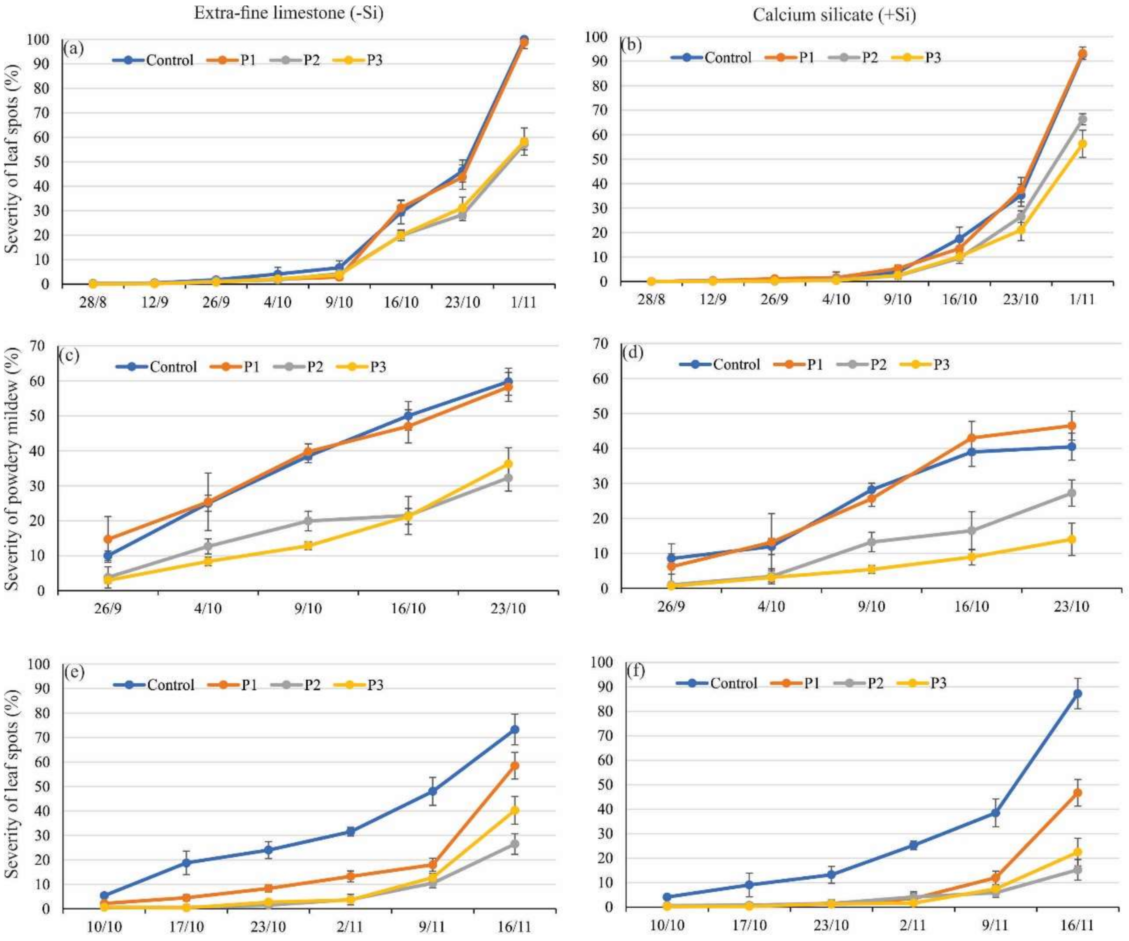

Figure S2. Leaf spot severity (a, b, e, and f) and powdery mildew severity (c and d) over time in barley plants of cultivar BRS Cauê grown in the absence (−Si) (a, c, and e) or presence (+Si) (b, d, and f) of silicon supply and either untreated (−F) or treated (+F) with fungicide during seasons I (a–d) and II (e and f) of Study II. P1, P2, and P3 represent the fungicide programs.

Table S3. Severity of powdery mildew and leaf spots in barley plants of cultivar BRS Cauê grown in the absence (−Si) or presence (+Si) of silicon (Si) during different growth stages (GS) in season I of Study II. Data are presented as means.

**Severity of Powdery mildew (%)**					
	GS30	GS35	GS45	GS55	GS61
−Si	10,00	25,00	38,45	50,00	59,75
+Si	9,48	11,50	28,20	38,98	40,50

**Severity of leaf spots (%)**					
	GS30	GS35	GS45	GS55	GS61
−Si	1,78	4,13	6,73	30,00	46,25
+Si	0,57	1,15	3,83	17,47	35,20

Table S4. Effect of silicon (Si) supply [absence (−Si) or presence (+Si)] and fungicide application [absence (−F) or presence (+F)] on the area under the disease progress curve (AUDPC), grain yield (kg ha^{-1}) and thousand grain weight (TGW, g) of barley BRS Cauê in the seasons I of the study II. Data are presented as mean.

**AUDPC of Powdery mildew**				
	Control	Fung. P1	Fung. P2	Fung. P3
−Si	992	995	480	418
+Si	696	728	316	166

**AUDPC of leaf spots**				
	Control	Fung. P1	Fung. P2	Fung. P3
−Si	1120	1060	682	701
+Si	863	873	600	513

**Yield (kg ha^{-1})**				
	Control	Fung. P1	Fung. P2	Fung. P3
−Si	1559	1618	2264	2115
+Si	1855	1788	2663	2993

TGW (g)				
	Control	Fung. P1	Fung. P2	Fung. P3
−Si	28,2	25,5	31,4	31,9
+Si	28,9	26,7	36,5	35,4

Table S5. Severity of powdery mildew and leaf spots in barley plants of cultivar BRS Cauê grown in the absence (−Si) or presence (+Si) of silicon (Si) during different growth stages (GS) in season II of Study II. Data are presented as means.

**Severity of leaf spots (%)**					
	GS30	GS35	GS45	GS55	GS61
−Si	5,38	18,75	24,00	31,50	48,00
+Si	4,13	9,08	13,25	25,25	38,50

Table S6. Effect of silicon (Si) supply [absence (−Si) or presence (+Si)] and fungicide application [absence (−F) or presence (+F)] on the area under the disease progress curve (AUDPC), grain yield (kg ha^{-1}) and thousand grain weight (TGW, g) of barley BRS Cauê in the seasons II of the study II. Data are presented as mean.

**AUDPC of leaf spots**				
	Control	Fung. P1	Fung. P2	Fung. P3
−Si	1216	553	217	288
+Si	982	295	150	159

**Yield (kg ha^{-1})**				
	Control	Fung. P1	Fung. P2	Fung. P3
−Si	2908	4295	4524	4076
+Si	3147	4250	5182	5134

TGW (g)				
	Control	Fung. P1	Fung. P2	Fung. P3
−Si	32,5	38,7	40,9	39,8
+Si	34,0	41,3	43,7	42,5
